# Biofeedback Therapy Combined with Traditional Chinese Medicine Prescription Improves the Symptoms, Surface Myoelectricity, and Anal Canal Pressure of the Patients with Spleen Deficiency Constipation

**DOI:** 10.1155/2013/830714

**Published:** 2013-07-28

**Authors:** Yi-Bo Yao, Yong-Qing Cao, Xiu-Tian Guo, Jin Yi, Hong-Tao Liang, Chen Wang, Jin-Gen Lu

**Affiliations:** Department of Anorectal Surgery, Longhua Hospital Affiliated to Shanghai University of Traditional Chinese Medicine, 725 South Wanping Road, Shanghai 200030, China

## Abstract

In order to observe the clinical therapeutic effects of Yiqi Kaimi Prescription and biofeedback therapy on treating constipation with deficiency of spleen qi, the 30 cases in the control group were given oral administration of Yiqi Kaimi Prescription, in combination with anus-lifting exercise; the 30 cases in the treatment group were given biofeedback therapy on the basis of the afore mentioned methods for the control group. The TCM symptom scores and anorectal pressures before and after treatment were observed and evaluated. There were significant differences in TCM symptom scores, anorectal pressure, and clinical recovery rate before and after treatment. In the treatment group, the total recovery rate was 86.66%, while in the control group it was 50%; there were significant differences between the two groups (*P* < 0.01). Yiqi Kaimi Prescription coupled with biofeedback therapy is clinically effective for treating constipation with deficiency of spleen qi, and thus this method is applicable for functional constipation with deficiency of spleen qi.

## 1. Introduction

Constipation, defined as having a bowel movement less than three times a week or the necessity of using laxatives more than three times a week [1], is a common problem with high prevalence and greatly affects the life quality of people [2]. The prevalence rates were 22.6% for constipation in the primary care clinic of the United States [3]. The prevalence of constipation was 14.1%–27% in the different ages of women in Australia [4]. In Asia, the chronic constipation affects 15% of Sri Lankan school children and adolescents [5]. It is also reported that up to 30% of preschool children in the eastern district of Hong Kong had constipation [6]. The prevalence of constipation of China is about 6%, which is substantially lower than that in the western countries [7]. However, a survey on people who were above 60 in Tianjin and Xi'an revealed that the incidence of chronic constipation reached 7.3%~20.39% [8]. With the aging of society, quickening pace of the modern life and the influencing of improper life habits (e.g., lack of fiber in food, irregular defecation, and subjective inhibition of desire for defecation), the incidence rate of this disease is increasing year by year.

Symptoms of constipation include infrequency of spontaneous defecation, difficulty initiating or completing bowel movement, rectal bleeding, pain, abdominal distention, and faecal incontinence [9]. It is known that the constipation was associated with multiple complications such as faecal impaction and bowel perforation [10]. The occurrence of constipation was related to many factors, so a single therapy will not achieve satisfactory effects. The chronic constipation may be caused by the diet deficient in fiber and fluids. It is reported that the moderate physical activity and increasing fiber intake were associated with substantial reduction in the prevalence of constipation in women [11]. However, the function of fiber in a diet for the chronic constipation was controversial. Although some patients may be helped by a fiber-rich diet, many patients with more severe constipation get worse symptoms when increasing dietary fiber intake [12]. There was also no evidence that constipation can successfully be treated by increasing fluid intake unless there is evidence of dehydration [13]. Functional limitations such as immobility and lack of exercise were other important reasons. A cross-sectional survey supported the notion that those who undertake more physical activity do have a lesser incidence of constipation [14]. The symptom of constipation was also a common adverse effect of many medications including analgesics, antidepressant drugs, iron salts, nonsteroidal anti-inflammatory drugs, and opiates. It is reported that factors other than opioid dose and physical functioning may be more important in contributing to constipation [15]. The afore-mentioned study suggested that the combination of many methods or therapies provides more possibilities for the diagnosis and cure.

TCM syndrome differentiation is of unique advantage, and hence it is widely accepted and applied in treating digestive tract diseases, which collect and analyze symptoms and signs to evaluate the overall conditions and to classify the pattern of maladjustment through determining the nature and location of the maladjustment [16]. It is reported that patients in the active treatment groups (standard and individualized Chinese herbal medicine) had significant improvement in bowel symptom scores when compared with patients in the placebo group [17]. The result of a systematic review suggested that traditional Chinese medicine interventions appear to be useful to manage constipation [18]. However, a research of European and Asian demonstrated that there was no clear consensus as to whether complementary therapies were beneficial, although the symptoms of many patients with inflammatory bowel disease were improved [19]. In general, the effects of traditional Chinese medicine therapy in treating the digestive system diseases were still controversial and need more well-designed studies.

Biofeedback, as a nonmedicinal and noninvasive therapy, has no side effects and can be used repeatedly, so it is regarded as the first choice for the clinical treatment of functional constipation. It is widely recognized that oral administration of Chinese medicine in combination with biofeedback therapy can be very effective for functional constipation. Our study aims at investigating the clinical therapeutic effects of Yiqi Kaimi Prescription and biofeedback therapy on treating constipation with deficiency of spleen qi, which could provide evidence for biofeedback therapy with traditional Chinese medicine prescription in treating diseases.

## 2. Methods

### 2.1. Clinical Data

The 60 patients were diagnosed as functional constipation at the department of proctology from June 2009 to May 2011. The two groups included 27 males and 33 females, averaging 40.28 ± 11.40 years old. There were no significant differences between the groups in gender, age, disease course, and scores (*P* > 0.05). The constipation symptoms of patients were determined by the Rome III diagnostic criteria [20]. The TCM diagnostic criteria for deficiency of spleen qi syndrome include the primary symptoms and secondary symptoms. The primary symptoms were dry stools resembling chestnuts, with desire for defecation yet without strength to complete it, shortness of qi, and pale tongue. The secondary symptoms were whitish complexion, lassitude with weakened qi, weary limbs with reluctance to speak, and weak pulse. Patients with 3 items of the primary symptoms or 2 items of the primary symptoms plus 2 items of the secondary symptoms could be determined.

### 2.2. Treatment Methods

The two groups were given Yiqi Kaimi Prescription before the treatment. The Yiqi Kaimi Prescription was composed of the Raw Astragalus (30 g), Atractylodes (15 g), Aitrus Aurantium (12 g), Almond (12 g), Radix Rehmanniae (15 g), and Rngelica (15 g). It was taken orally twice a day, and four weeks made up a disease course. Before the treatment, the anorectal pressure was measured. The treatment group was given oral Yiqi Kaimi Prescription in combination with biofeedback treatment as well as anorectal muscle electrical testing. The control group was given oral Yiqi Kaimi Prescription in combination with anus-lifting exercise. 

The specific procedures and main indexes of anorectal pressure test were as follows. The urine and feces were evacuated before the test. And the whole process should be explained in detail to the patient. At first, put a pressure-measurement tube into the anus at 6 cm in depth, and then tell the patient to imitate the effort to defecate, which lasted for 5~10 seconds. After 5 seconds of rest, ask the patient to contract his anus for 5~10 seconds; then adjust the depth of the tube to 2 cm, and tell the patient to rest for 30~60 seconds; afterwards repeat the previous procedures; use a syringe to rapidly inject 10 mL of gas into the anus at the 2 cm position, then discharge the gas 1 or 2 seconds later, and meanwhile observe the pressure difference and if the pressure curve would present double phase waves; inflate 50 mL of gas into the saccule and at the same time tell the patient to imitate the effort to defecate, which should last for 5~10 seconds; after 30~60 seconds of rest, inflate the saccule with a syringe slowly and evenly, and meanwhile record the initial feels, initial pressing and intense pressuring, of the patients. 

For specific procedures of biofeedback, The patient should lie on his or her back and bend the body until the upper half and the lower half formed a 120° angle, and then the doctor put the measuring electrode of the biofeedback equipment into the anus and conducted Glazer assessment (test when relaxing the anus for 60 seconds, rapidly contracting it for 5 times/relaxing for 10 seconds, contracting for 10 seconds/relaxing for 10 seconds, persistently contracting for 60 seconds, and posterior baseline for 60 seconds). 

Above 2 uv at the vagina and above 4 uv at the anus when relaxing, first carry out pelvic floor muscle multimedia training, electrostimulation on the pelvic floor nerves and muscles, myoelectricity triggering electric stimulation, and template Kegel training. If the pelvic floor muscles contract rapidly and the index is lower than 37.5 at the vagina and lower than 70 uv at the anus, yet there is no increase of resting value, the electrostimulation on the pelvic floor nerves and muscles, myoelectricity triggering electric stimulation, and template kegel training should be carried out first once a day and 20 min a time for 3 treatment courses; anorectal muscle electrical testing was performed every six days; a treatment course includes 10 days. The changes of TCM symptoms after treatment and retest of the anorectal pressure will be observed.

### 2.3. Therapeutic Criteria

The therapeutic criteria for constipation are made according to the Guiding Principles for Clinical Research of New Traditional Chinese Medicines stipulated by China's Ministry of Health in 1993 [21]. 

### 2.4. Statistical Analysis

The comparisons of curative rate and disappearance rate of positive transmission test were analyzed by *X* ^2^ test. The clinical symptom scores and life quality scores were processed by *t*-test or analysis of variance. 

## 3. Results

In this study, it was found that patients with deficiency of spleen qi cannot be simply diagnosed as slow transmission constipation (STC). The weakened intestinal motive power was often accompanied by uncoordinated contraction of the pelvic floor muscles and interferences of psychological factors during defecation. Before the treatment, the survey on the age and gender baselines of the patients found that the average onset age of the patients with deficiency of spleen qi was 40.28 ± 11.40 years old. Constipation with deficiency of spleen qi was common in old people, and females account for a large proportion, which may be due to the influence of menopause and hormone levels.

**Figure 1:**
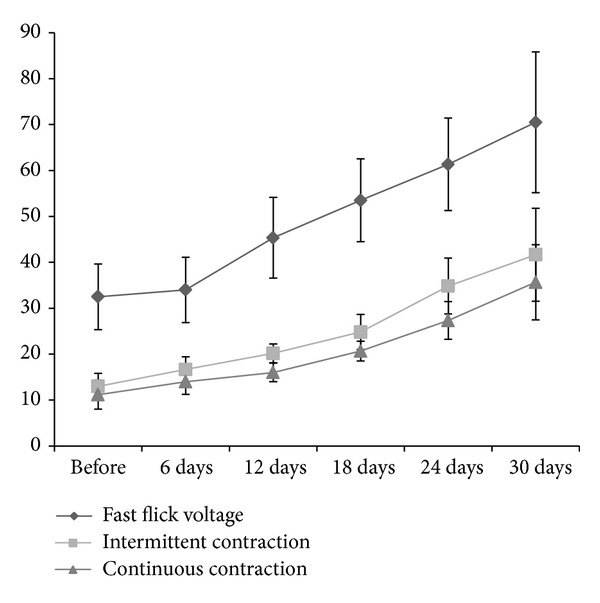
The trends of fast flick voltage, intermittent contraction, and continuous contraction within 30 days in the treatment group. The anorectal muscle electricity of patients in the treatment group continued to rise in the six-time detection, including the fast flick voltage and intermittent and continuous contraction voltages, which suggested that the strength has been significantly improved.

**Figure 2:**
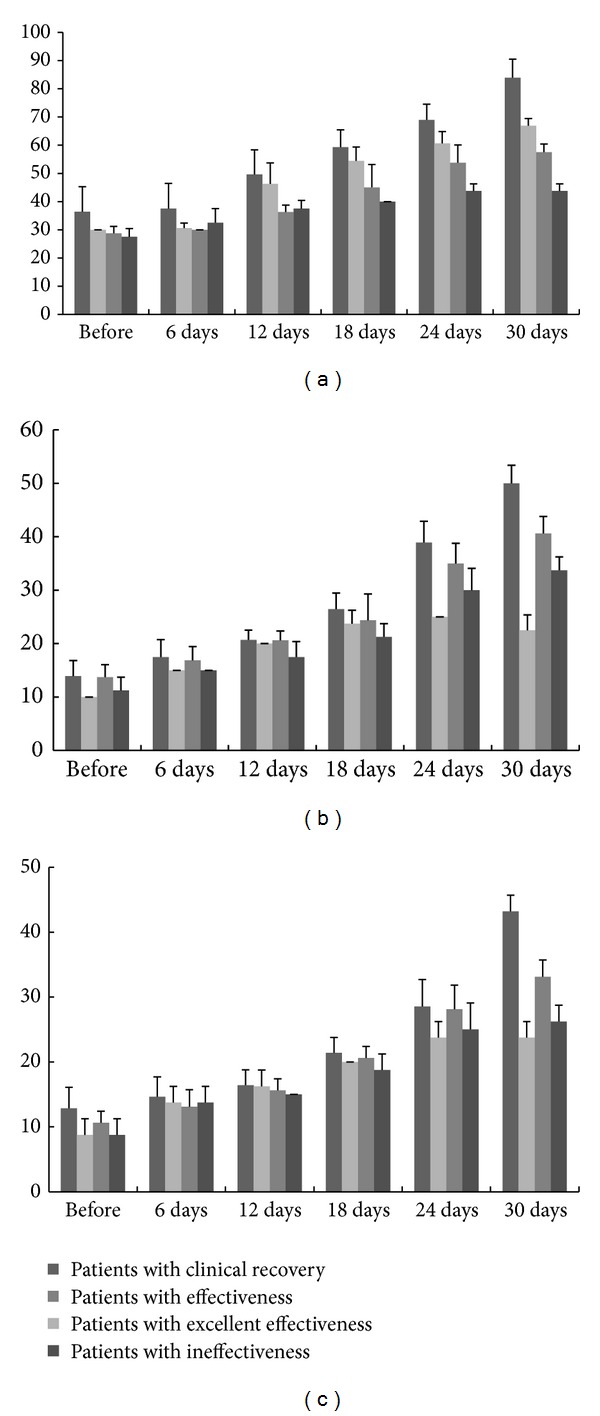
The comparison of the fast flick voltage, intermittent contraction voltages, and continuous contraction voltages among the groups of patients with clinical recovery, patients with excellent effectiveness, patients with effectiveness, and patients with ineffectiveness. (a) The trends of the fast flick voltage among the 4 groups. (b) The trends of the intermittent contraction voltages among the 4 groups. (c) The trends of the continuous contraction voltages among the 4 groups.

The results showed that the constipation symptoms, such as difficult defecation, endless and dilatation felling, and abdominal distension, had been significantly improved (*P* < 0.05; see Table 1). The defecation time was also significantly shortened after treatment from 2.1 min to 0.96 min, which had a significant difference when compared to that of the control (*P* < 0.05). After biological feedback therapy, the anorectal muscle electricity of patients in the treatment group was significantly improved (*P* < 0.05); at the same time, anorectal muscle electricity continues to rise in the six-time detection; the fast flick voltage and intermittent and continuous contraction voltages were significantly elevated, which suggested that the strength has been significantly improved. The change of the resting muscle electricity was not obvious, which suggested that the impact of the biofeedback treatment on the pelvic floor muscle was weak. However, the resting muscle electricity of the 30 patients in the treatment group was tested every 6 days. The result showed that the pelvic floor muscles get more training; the myo-electrical value and the muscle endurance were significantly improved at the same time (see Figure 1). Meanwhile, after biofeedback therapy combined with traditional Chinese medicine prescription, the fast flick voltage, and intermittent contraction voltages, continuous contraction voltages of 14 patients with clinical recovery were greatly improved after 3 medical courses. However, the fast flick voltage, intermittent contraction voltages, and continuous contraction voltages of 4 patients with ineffectiveness were not significantly changed, which may be related to the change of myoelectricity within 30% (see Figure 2).

The resting pressure of the internal sphincter, pressure of the external anal sphincter, and maximum systolic pressure significantly increased to some degree (*P* < 0.05; see Table 2). The contradictory contraction of the pelvic floor muscles during defecation was addressed; some patients had improved rectal sensory function, coordination between the rectum's motive power and the relaxation of the anus, and establishment of correct defecation. In both groups, there was no significant difference in the initial threshold values before and after treatment (*P* > 0.05). In the treatment group, there was significant difference in the maximum threshold values before and after treatment (*P* < 0.05). This indicated that biofeedback can improve the rectal sensory function; there was marked change in the treatment group after the treatment, indicating that the anus-lifting exercise lacks an effective nervous feedback-circuit training mechanism. Without regular and quantized training, the therapeutic effects were often unsatisfactory. However, in the treatment group, the biofeedback treatment coupled with electric stimulation can effectively improve the sensibility of the lower portion of the rectum, which may be due to the reparation of damaged nerves. However, the sample size of the study was small; the relationships among the conditions of the pelvic floor electromyography, the symptoms, and the anorectal pressure functional change were still unclear and need further research.

In general, the total effective rate of the treatment group was 86.66% (26 cases; see Table 3), the clinical recovery rate was 46.7% (14 cases), the excellent effectiveness rate was 26.66% (8 cases), the effectiveness rate was 13.33% (4 cases), and the ineffectiveness rate was 13.33% (4 cases).

## 4. Discussion

According to Rome III, functional constipation can be divided into three types: slow transmission constipation (STC), outlet obstructive constipation (OOC), and mixed pattern constipation [22]. Clinically the symptoms of functional constipation were various, which may differentiate STC from OOC. However, the treatment with clear target received unsatisfactory effects. Some patients had atypical symptoms of STC or OOC, long histories of drug reliance, and too many accompanying diseases and symptoms, which affected the diagnosis and treatment of constipation. The disease differentiation and syndrome differentiation of TCM had a unique advantage in the treatment of constipation. The diagnosis was made through examining the symptoms and signs of the patients as well as the anorectal pressure and made by combining TCM disease differentiation and syndrome differentiation [23]. After the diagnosis, the disease can be treated by traditional Chinese medicine coupled with modern therapeutics, and the effects were often satisfactory. 

TCM had a unique advantage in treating functional constipation [24]. It approached the disease from overall regulation, emphasized on the root cause, and treated it in view of time, locality, and individuality [25]. Flexible in medication, it took into account both the primary and the secondary aspects and treated the disease and syndrome simultaneously, thus fully playing the role of individualized treatment. Biofeedback training was a new psychological treatment developed on the basis of behavior therapy [26]. It was based on Kegel training and aimed at directing the patient to correctly exercise the pelvic floor muscles, hence changing the measurable physiological parameters, strengthening the contracting function of the pelvic floor muscles, and achieving the best effects for excises of pelvic floor muscles [27]. The main symptoms of patients with spleen deficiency constipation were the decrease of both the intestinal motility and the anal sphincter muscle power, un-coordination of the pelvic floor muscle movement. The prescription was able to regulate the qi activity, improve intestinal motility, and promote the power of anal sphincter muscle. On the other hand, biofeedback therapy could improve the pelvic floor muscle movement through the enhancement of the anal sphincter muscle strength, which improved the symptoms of spleen deficiency constipation.

The disease location of constipation was in the large intestine, but it was also closely related to the viscera, channels, qi, blood, fluids, and emotions. It was controlled by the spleen, so if the spleen qi was sufficient, the defecation will be normal; if the spleen qi was deficient, the transportation will be abnormal, or the qi transformation will be inadequate, leading to malfunction of transformation and, eventually, constipation. The root of constipation (deficiency of spleen pattern) was qi stagnation and/or spleen deficiency, and the branch was accumulation of heat. Unsmooth flow of qi leaded to the obstruction of the large intestine, or deficiency of qi failed to promote transportation, also leading to constipation. Qi deficiency and qi stagnation were often mixed and interpromoted, thus making the disease more refractory. In brief, unsmooth qi transformation, stagnated qi movements, and insufficient qi and fluid were the fundamental causes of this disease.

Deficient spleen qi failed to promote transportation, so the intestinal movements and transportation of water and food will slow down, leading to prolonged retaining of food dregs in the intestinal tract and excessive absorption of water, characterized by dry, hard stools like chestnuts. The dysfunction of the spleen and stomach in digestion gives rise to abnormal ascending of food essences and abnormal descending of turbid things, and consequently the promoting ability of the intestines will be weakened. The spleen and stomach were the prenatal foundation, so their malfunction will cause disordered circulation of qi and blood in the whole body, marked by whitish complexion, lassitude with weakened qi, weary limbs with reluctance to speak, weak pulse, and decreased muscle strength, as well as asynchrony of abdominal muscle strength and pelvic floor muscle group in varying degrees. 

Yiqi Kaimi Prescription was effective for constipation due to deficiency of spleen qi [28]. It used Raw Astragalus and Atractylodes as the monarch herbs and used Aitrus Aurantium and Almond to regulate qi so as to dredge both the upper orifices and lower ones and promote the transportation of the large intestine. If coupled with a small quantity of Radix Rehmanniae, they can moisten the intestinal tract, nourish the middle energizer, and regulate qi activity. Once the qi was replenished, it will regain its original abilities, break the stagnations, and remove the obstructions. Through nourishing qi, ascending the clear, and descending the turbid, as well as steaming the fluids, the yin will be nourished, so will the fluids. The fluids and qi were interchanged and mutually moistened. All of these factors worked together so as to effectively improve the intestinal motive power and restore the transportation of the stomach and intestines. 

The anorectal pressures before and after the treatment were measured. Although the technique cannot give a direct view of the structural disorder of the gastrointestinal tract, it can objectively describe the functional disorder of the gastrointestinal tract [29]. It was one of the main methods which can reveal whether the constipation was due to structural disorder or due to functional disorder and a way to classify chronic constipation. It can also help us understand, quantize, and evaluate the defecation function of the anal canal and rectum, thus providing pathological and physiological foundations for the researches into anorectal disorders such as abnormal defecation [30]. The changes of sensory threshold value also played a critical role in the occurrence and development of constipation [31]. It can provide objective foundation for the contradictory contraction of the anus and abdominal muscles and therefore guide clinical treatment. Patients with constipation due to deficiency of spleen qi often had anal laxity and relatively low resting pressure of the internal sphincter, pressure of the external anal sphincter, and maximum squeezing pressure. When making an effort to defecate, the patients may present with inadequate increase of rectal pressure, weak abdominal strength, and contradictory contraction of anal sphincter, though such occasions are rare. There was also a slight increase of the initial sensory threshold value of the rectum, initial defecation threshold value, and maximum toleration value of the rectum. Besides, in patients with constipation due to spleen deficiency, there may be damages of the internal motor nerves, which require further studies for confirmation.

For some patients with colon transportation disturbances without apparent symptoms of outlet obstruction, their situations can also be improved through biofeedback treatment [32]. This indicated that biofeedback can promote defecation at the same time of improving anorectal sensibility. However, the effects of placebo and psychological factors cannot be excluded. The patients in the control group performed anus-lifting exercise, yet without effective guidance and electric stimulation. The effect was not obvious, and the effect of the treatment group was far better than that of the control group. Traditional Chinese medicine coupled with biofeedback treatment can effectively improve the intestinal motive power, with marked improvement of TCM symptoms and alleviation of exhaust gas and abdominal distension. Biofeedback treatment was used to actively train the contraction of pelvic floor muscles so as to strengthen them, improve their supportive force and prevent prolapse and slackness of the pelvic floor. It can display the muscular electric activities by electrodes inserted into the rectum or abdomen. The physiological signals in these positions can be amplified and transformed into visible ones such as sound, lights, and graphs, which will be fed back to the patients. The patients trained themselves on the basis of these signals, and gradually developed conditioned reflex, and learned to voluntarily control the contraction of pelvic floor muscles. For patients with reduced sensitivity, the stimulation will be increased mainly by stimulus wave produced by microcurrent, which changed the central nervous system indirectly and regulated the pelvic floor nervous system. In this study, the treatment group received three courses of biofeedback treatment in addition to traditional Chinese medicine. 

Multipattern combined treatment of functional constipation was of great significance. Constipation cannot be explained by monism, so medication should be combined with biofeedback so as to regulate the intestinal motive power and strengthen the coordination of pelvic floor muscle groups. The clinical therapeutic effects were often satisfactory.

## Figures and Tables

**Table 1:** The score before and after treatment.

	Treatment group		Control group	
	Before	After	Before	After
Difficult defecation	2.06 ± 0.37	1.06 ± 0.58^∗#^	2.07 ± 0.37	1.10 ± 0.30*
Fecal character	2.07 ± 0.42	0.79 ± 0.38^∗#^	2.10 ± 0.45	1.34 ± 0.45^∗#^
Defecation time (min)	2.10 ± 0.30	0.96 ± 0.18^∗#^	1.87 ± 0.58	1.21 ± 0.41^#^
Endless and dilatation felling	2.03 ± 0.31	1.57 ± 0.50^∗#^	1.90 ± 0.54	1.17 ± 0.38^#^
Frequency	2.14 ± 0.48	0.86 ± 0.48*	2.10 ± 0.45	0.82 ± 0.43*
Abdominal distension	2.17 ± 0.46	0.87 ± 0.35^∗#^	1.90 ± 0.55	1.53 ± 0.51^#^

Data were shown as mean ± SD (*n* = 30). Statistical comparisons were made by the Student's *t*-test. *indicates *P* < 0.05, compared to that of before; ^#^Indicates *P* < 0.05, compared to that of control.

**Table 2:** The rectum pressure before and after treatment.

	Treatment group		Control group	
	Before	After	Before	After
Anal rest pressure	38.23 ± 13.14	53.70 ± 10.07^∗#^	30.90 ± 14.90	39.23 ± 10.45^#^
Maximum systolic pressure	100.48 ± 22.55	128.83 ± 23.38^∗#^	88.97 ± 20.45	95.53 ± 12.19^#^
Rectal initial threshold	39.73 ± 9.26	35.76 ± 5.50	37.43 ± 6.67	36.53 ± 5.30
Rectal maximum threshold	246.47 ± 46.50	225.13 ± 42.71^∗#^	218.83 ± 52.50	219.93 ± 45.80

Data were shown as mean ± SD (*n* = 30). *indicated *P* < 0.05, compared to that of the control, ^#^Indicated *P* < 0.05, compared to that of the before treatment.

**Table 3:** The comparison of the clinical effects.

Groups	Cases	Total effective rate	Clinical recovery rate	Excellent effectiveness rate	Effectiveness rate	Ineffectiveness rate
Treatment group	30	26 (86.66%)	14 (46.7%)	8 (26.66%)	4 (13.33%)	4 (13.33%)
Control group	30	15 (50.00%)	2 (6.66%)	5 (16.67%)	8 (26.66%)	15 (50.00%)
